# Electrospun fibers enhanced the paracrine signaling of mesenchymal stem cells for cartilage regeneration

**DOI:** 10.1186/s13287-021-02137-8

**Published:** 2021-02-03

**Authors:** Nurul Dinah Kadir, Zheng Yang, Afizah Hassan, Vinitha Denslin, Eng Hin Lee

**Affiliations:** 1 Department of Orthopaedic Surgery, Yong Loo Lin School of Medicine, National University of Singapore, NUHS Tower Block, Level 11, 1E Kent Ridge Road, Singapore, 119288 Singapore; 2 Tissue Engineering Program, Life Sciences Institute, National University of Singapore, DSO (Kent Ridge) Building, #04-01, 27 Medical Drive, Singapore, 117510 Singapore

**Keywords:** Mesenchymal stem cells, Fiber orientation, Paracrine signaling, Mechanotransduction pathway, Cartilage repair

## Abstract

**Background:**

Secretome profiles of mesenchymal stem cells (MSCs) are reflective of their local microenvironments. These biologically active factors exert an impact on the surrounding cells, eliciting regenerative responses that create an opportunity for exploiting MSCs towards a cell-free therapy for cartilage regeneration. The conventional method of culturing MSCs on a tissue culture plate (TCP) does not provide the physiological microenvironment for optimum secretome production. In this study, we explored the potential of electrospun fiber sheets with specific orientation in influencing the MSC secretome production and its therapeutic value in repairing cartilage.

**Methods:**

Conditioned media (CM) were generated from MSCs cultured either on TCP or electrospun fiber sheets of distinct aligned or random fiber orientation. The paracrine potential of CM in affecting chondrogenic differentiation, migration, proliferation, inflammatory modulation, and survival of MSCs and chondrocytes was assessed. The involvement of FAK and ERK mechanotransduction pathways in modulating MSC secretome were also investigated.

**Results:**

We showed that conditioned media of MSCs cultured on electrospun fiber sheets compared to that generated from TCP have improved secretome yield and profile, which enhanced the migration and proliferation of MSCs and chondrocytes, promoted MSC chondrogenesis, mitigated inflammation in both MSCs and chondrocytes, as well as protected chondrocytes from apoptosis. Amongst the fiber sheet-generated CM, aligned fiber-generated CM (ACM) was better at promoting cell proliferation and augmenting MSC chondrogenesis, while randomly oriented fiber-generated CM (RCM) was more efficient in mitigating the inflammation assault. FAK and ERK signalings were shown to participate in the modulation of MSC morphology and its secretome production.

**Conclusions:**

This study demonstrates topographical-dependent MSC paracrine activities and the potential of employing electrospun fiber sheets to improve the MSC secretome for cartilage regeneration.

**Supplementary Information:**

The online version contains supplementary material available at 10.1186/s13287-021-02137-8.

## Background

Chondral lesions of the knee are a significant cause of pain and disability in patients with traumatic injuries. Cartilage defects can accelerate the wear of the knee joint due to its poor self-regenerative capacity and, if not addressed, will predispose the joint to the development of osteoarthritis. Current clinical treatments have inconsistent therapeutic benefits as indicated by the formation of neocartilage with inferior properties [1–3]. Given that inflammation is a hallmark of injured cartilage, in which pro-catabolic activities in chondrocytes lead to extracellular matrix (ECM) homeostatic imbalance [4], an effective approach for cartilage regeneration should address the regulation of the inflammatory environment in the injured knee joint, while promoting recruitment, proliferation, maturation, and survival of the endogenous chondrocytes and progenitor stem cells.

Mesenchymal stem cells (MSCs) hold significant promise for cell-based therapies in cartilage tissue engineering due to their relative ease of availability, high proliferative capacity, and chondrogenesis potency [5]. The concept that MSCs become engrafted and subsequently differentiate into chondrocytes at the defect site [6] has been reconsidered with recent evidence showing that the paracrine secretion of trophic factors, namely the MSC secretome, plays an important role in the overall cartilage tissue regeneration [7]. MSC secretome contains a plethora of biologically active factors, ranging from cytokines, cytokine receptors, growth factors, enzymes, enzyme inhibitors, peptides, and miRNAs, as soluble proteins, or packaged in the extracellular vesicles [8]. MSC secretome in the form of conditioned media (CM) [9], or its isolated components, such as exosomes [10–13] exhibit cartilage regenerative potential in osteochondral defects and in an osteoarthritis model. The demonstrated therapeutic benefit of MSC secretome in cartilage regeneration opens up the opportunity for a cell-free therapy. However, secretome-induced cartilage regeneration often derived MSC secretome from tissue culture plate (TCP)-cultivated MSCs. The absence of a physiological microenvironment in TCP culture would not have provided the optimum conditions for MSC functionality and paracrine secretion. Accordingly, high doses of secretome / exosomes have to be employed to exert their therapeutic efficacy [10, 11, 14, 15]. Thus, exploration for alternative MSC culturing platform is warranted to improve the efficacy of MSC secretome for therapeutic application.

Local microenvironment plays a vital role in influencing the MSC paracrine production and activities [16]. Presence of growth factors and cytokines activate MSC inflammation modulatory response by altering MSC paracrine production [17, 18]. Anti-inflammatory therapeutic effect of MSCs could also be influenced by cell-cell interaction [19], in which the delivery of MSCs in spheroid reduced macrophage inflammatory phenotype. Further, biomaterials that promote cell aggregation improved MSC paracrine function on myoblasts [20]. Extracellular environment, such as biochemical, mechanical, and topographical cues affect cell-matrix interaction and the activation of mechanotransduction signaling, influence not only MSC proliferation and differentiation [21, 22], but also have an impact on MSC secretome. This includes the enhancement of MSCs’ pro-angiogenic activities by glycine-histidine-lysine peptides in alginate hydrogels [23]; the influence on MSC secretome repertoire by scaffold stiffness [24] and surface topography of substrate, which has been demonstrated to have a paracrine therapeutic effect for numerous disease conditions [20, 25, 26].

**Fig. 1:**
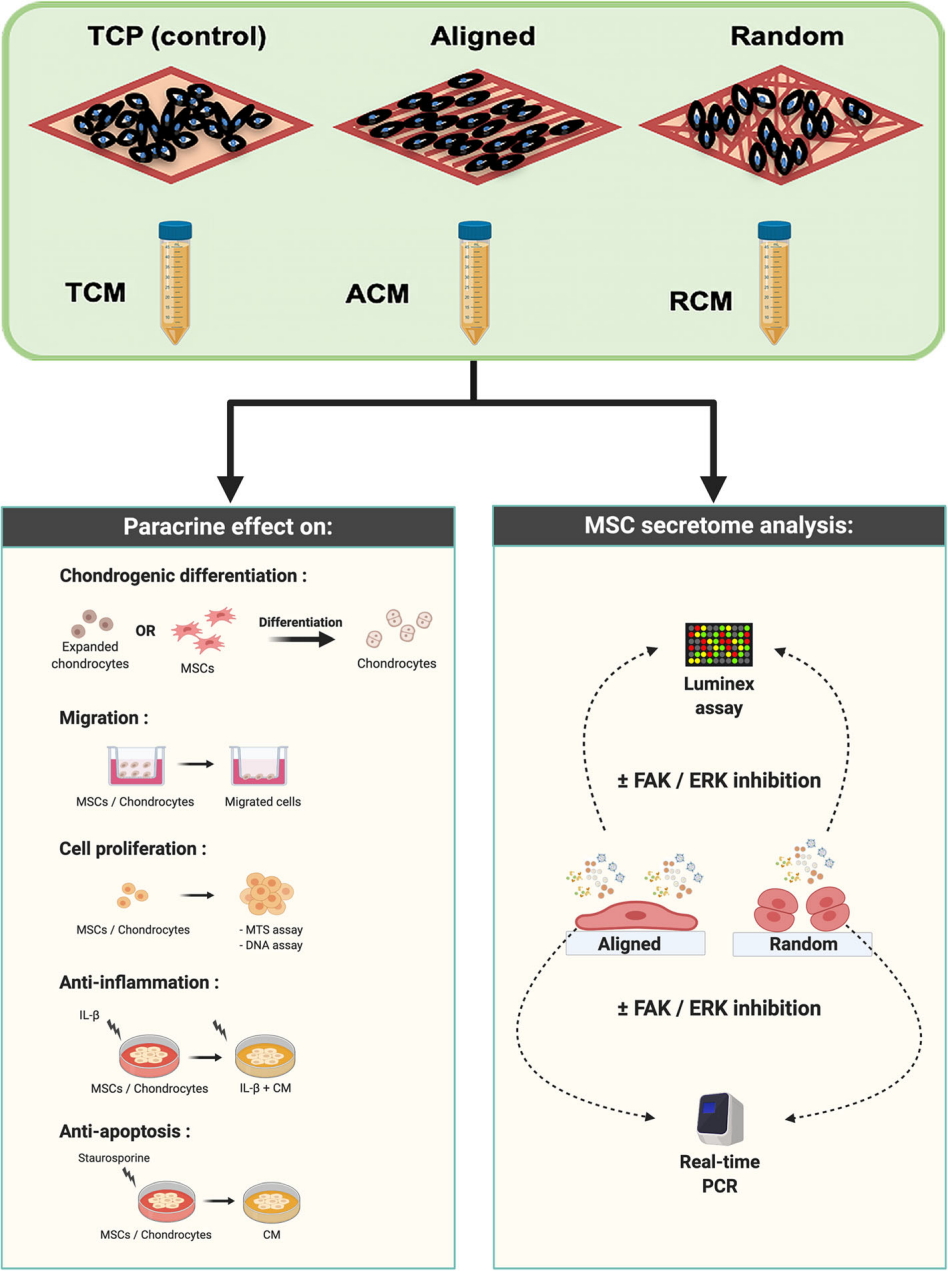
Illustration of CM generation, functional studies of the CM, and investigation of mechanotransduction pathways relative to topographical-dependent MSC secretome. Created with BioRender.com

In this study, we employed a poly-L-lactide-co-ε-caprolactone (PLCL) electrospun fiber sheets with aligned and randomly oriented fibers as the topographically defined culture platform to generate MSC-CM. The fiber material was chosen due to its biocompatibility, elastic properties and its ability to promote MSC chondrogenesis [27]. In particular, the fiber orientation mimics the natural extracellular matrix environment and induces morphological changes in MSCs. The distinct cell-to-fiber contact and traction force has been found to provide cues to modulate cell activities, which includes promoting MSC differentiation [25], cell migration and proliferation [20], as well as inflammatory modulation [25, 26]. In vitro functional studies were performed on chondrocytes and MSCs to assess the influence of fiber orientation in affecting cartilage regeneration through the migratory, proliferative, chondrogenic, anti-inflammatory, and anti-apoptotic properties of MSC secretome, in comparison to the conventional TCP-generated secretome (Fig. 1). The underlying signaling pathways involved in affecting the secretion of MSC paracrine factors with respect to the distinct fiber orientation were also investigated.

## Methods

### Fabrication of electrospun fiber sheets

Ten percent (w/v) PLCL (Corbion, Netherlands) polymer solution was prepared in 1,1,1,3,3,3-hexafluoro-2-propanol (Sigma-Aldrich) for electrospinning. Briefly, the polymer solution was loaded into a syringe and ejected at 1.0 ml/h with a voltage of 15 kV. The aligned and randomly oriented fibers were collected on a rotating collector at the speed of 3000 and 300 rpm, respectively. The images of the fiber sheets were captured using a scanning electron microscope (SEM) (FEI Quanta 650 FEG) to assess the topographical features, fiber diameter, scaffold thickness, and percentage of porosity via ImageJ software. The mean diameter of fibers was determined by selecting 100 fibers randomly in each sample (*n* = 6), while the thickness of fiber sheets was measured from its cross-sectional view under SEM. The percentage porosity was quantified based on the percentage of area fraction in each sample, while orientation of fibers was determined via the Orientation J plugin in the ImageJ software.

### MSC and chondrocyte cell culture

Human bone marrow-derived MSCs from three donors were purchased from RoosterBio Inc. (Frederick, MD). MSCs were expanded in MSC high-performance media (RoosterBio Inc.) until 70–80% confluency at 37 °C in 5% CO_2_ atmosphere and were used at passage 5 for all the experiments.

Chondrocytes were isolated from the femoral condyle of healthy pig articular cartilage (6–9 months old). Briefly, cartilage tissues were digested with TrypLE (Life Technologies, USA) and 0.25% (w/v) type II collagenase solution (Life Technologies) overnight before chondrocytes were isolated and expanded in low glucose (LG)—DMEM (Life Technologies), 10% FBS (HyClone), and 1% glutamax at 37 °C in 5% CO_2_ atmosphere. Chondrocytes were used at passage 1 for all further experiments.

### Fluorescence cell imaging

To examine the cell morphology on the fiber sheets, MSCs attached to fibers for 24 h were fixed in 10% formalin and then incubated with TRITC-conjugated phalloidin (1:1000; Life Technologies), before mounted with slow-fade gold fluorescent mounting medium containing DAPI (Life Technologies). Images of stained cells were captured using an Olympus IX81 inverted fluorescence microscope.

### Preparation of MSC conditioned media

MSCs were cultured on three different substrates: tissue culture plate (TCP), aligned fiber sheets, and randomly oriented fiber sheets. MSC confluency was allowed to reach 60–70% in standard culture media before replaced with serum-free LG-DMEM. The CM was collected 24 h later as TCP- (TCM), aligned fiber- (ACM), and randomly oriented fiber-generated conditioned medium (RCM). The collected CM was centrifuged at 200×*g* for 5 min, followed by 500×*g* for 10 min to remove dead cells and cellular debris. All CM were concentrated 10× by high centrifugation force at 4000×*g* in 4 °C, using a protein concentrator with a molecular weight cut-off of 3 kDa (Thermo Fisher Scientific, USA). To ensure that all concentrated CM in different culture platforms were generated from the same number of cells, the CM was normalized to the total number of cells laden on TCP and fiber sheets. In subsequent functional studies, the 10× concentrated CM was diluted to 1× concentration (non-concentrated CM) using the appropriate assay media.

### Inhibition studies of mechanotransduction pathways

The FAK and ERK pathways were targeted for inhibition study. MSCs were pre-incubated with either 20 μM of PF573228 (FAK inhibitor; Sigma-Aldrich) or 70 μM of PD98059 (ERK inhibitor; Cell Signaling Technology) for 1 h before seeded on the TCP, aligned or randomly oriented fiber sheets in expansion media. Western blot analysis of phosphorylated FAK (p-FAK) and ERK (p-ERK) expression was performed on MSCs after 24 h of seeding, while mRNA and its secretory protein were analyzed after a further incubation of 24 h in serum-free media.

### Cell migration

Cell migration was assessed using a transwell culture system (8 μm pore size; Millipore, Germany). Low-serum culture media (DMEM with 0.5% FBS) and standard culture media (DMEM with 10% FBS) were used as negative and positive control, respectively. In brief, 5 × 10^4^ chondrocytes or 3 × 10^4^ MSCs in low-serum culture medium were seeded in the upper chamber, while the differential CM, low-serum or standard culture medium were placed in the lower chamber. Following 16 h of incubation, migrated cells on the underside of the filters were fixed with 10% formalin and stained with hematoxylin and eosin (Sigma-Aldrich, USA). For each filter samples, cells at × 100 magnification from 5 randomly selected fields were counted to determine the number of migrated cells.

### Cell proliferation

Cell proliferation was determined by measuring the metabolic activity and total DNA content of the treated cells. MSCs and chondrocytes were cultured in 96-well plates at a seeding density of 2 × 10^3^ and 5 × 10^3^ cells/well, respectively. Cells were then treated with low-serum media in the presence or absence of differential CM. Low-serum media were used to limit the basal cell proliferation capacity as serum contains growth factors that can stimulate proliferation of cells. Metabolic activity was measured by MTS (3-(4,5-dimethylthiazol-2-yl)-5-(3-carboxymethoxyphenyl)-2-(4-sulfophenyl)-2H-tetrazolium) assay kit (Promega, USA). The percentage of cell proliferation was determined by normalizing the absorbance of MTS in each sample against the control group at their respective time point, which was set at 100%. Total cellular DNA quantification was determined using the Quant-iT PicoGreen dsDNA assay kit (Life Technologies).

### Chondrogenic culture

The chondrogenic 3D-pellet culture of MSCs or chondrocytes was induced in a standard chondrogenic media supplemented with differential CM, in the presence or absence of TGF-β3 (R & D Systems, Canada). Briefly, 2.5 × 10^5^ MSCs or chondrocytes were centrifuged to form pellets and cultured in chondrogenic differentiation media, which composed of high-glucose DMEM supplemented with 10^− 7^ M dexamethasone (Sigma-Aldrich), 1% ITS-Premix (Becton-Dickinson, San Jose, CA), 50 μg/mL ascorbic acid, 1 mM MEM sodium pyruvate, 4 mM proline, 100 unit/mL of penicillin, 100 μg/ml of streptomycin, and 1% (v/v) glutamax. TGF-β3 at 10 ng/ml and 2 ng/ml was used to induce chondrogenic differentiation of MSCs and re-differentiation of chondrocytes, respectively.

### Inflammatory induction of chondrocytes and MSCs

The IL-1β concentration used for inflammatory induction in chondrocytes (5 ng/ml) and MSCs (0.5 ng/ml) were based on the IL-1β titration study (Additional file 1: Figure S1). Samples without IL-1β treatment served as the non-inflammation (Negative) control, while samples treated with only IL-1β served as the inflammation (Positive) control.

In the chondrocyte inflammation study, cartilage tissue was generated from re-differentiation of chondrocyte pellets in the standard chondrogenic media with TGF-β3 (10 ng/ml) for a week. Inflammation was then induced with IL-1β (5 ng/ml) in the absence of TGF-β3 for subsequent 7 days, in the presence or absence of CM. Pellets were harvested 14 days post-chondrogenic treatments to evaluate the conservation of cartilage ECM at both mRNA and protein levels.

For the MSC inflammation study, TGF-β3 (10 ng/ml)-induced chondrogenic differentiation of MSC pellets were treated with IL-1β at 0.5 ng/ml in the presence or absence of CM. Pellets were harvested at day 7 and day 21 to evaluate the MSC chondrogenic efficiency at both mRNA and protein levels.

### Apoptosis analysis

The anti-apoptotic potential of CM on MSCs and chondrocytes was assessed using a staurosporine-induced apoptosis model. MSCs and chondrocytes were cultured in 96-well plates at a seeding density of 2 × 10^3^ and 5 × 10^3^ cells/well, respectively. To induce apoptosis, cells were treated with 1 μM staurosporine (Sigma-Aldrich) in a standard culture media for 3 h, before replaced with a low-serum medium in the presence of differential CM. Cells without exposure to staurosporine served as the negative control, while cells exposed to staurosporine with no further treatment with CM served as the apoptotic (Positive) control. After 24 h of treatment, the apoptotic activity of cells was measured using the Caspase-Glo® 3/7 luminescent assay (Promega) on TECAN Infinite M200 plate reader.

### Characterization of MSC secretory factors

MSC secretory factors were quantitated using a customized Luminex assay (Merck Millipore). Luminex MAGPIX fluorescent imager was used to determine the fluorescent intensities of targets, and the protein concentrations were then extrapolated from the standard curve generated via Luminex xPONENT software.

### Real-time PCR analysis

Total RNA was extracted using the RNeasy® Mini Kit (Qiagen, Germany). The quality and concentration of RNAs were determined by NanoDrop (NanoDrop Technologies, Wilmington, DE). Reverse transcription was performed with 100 ng total RNA using iScriptTM cDNA synthesis kit (Bio-Rad, USA). Real-time PCR was performed using the Power SYBR® green PCR master mix on ABI Step One Plus Real-time PCR System (Applied Biosystems, Life Technologies) at 95 °C for 10 min, followed by 40 cycles of amplifications, consisting of 15 s denaturation at 95 °C and 1 min extension at 60 °C. Primer sequences of targeted genes were listed in Additional file 2: Table S1. The expression levels of targeted genes were normalized to glyceraldehyde-3-phosphate dehydrogenase (GAPDH) as the reference gene. Fold changes were then calculated using the 2^−ΔΔCt^ formula with reference to the undifferentiated MSCs.

### Histology staining

Paraffin-embedded tissue sections were stained for proteoglycan with 0.1% Safranin O solution (Acros Organics, USA) and counterstained with 0.1% fast green and hematoxylin (Sigma-Aldrich). For identification of type II collagen and ADAMTS-5, tissue sections were incubated with type II collagen monoclonal antibodies (0.2 mg/ml Clone 6B3; Chemicon, Inc., USA) or ADAMTS-5 polyclonal antibodies (0.25 mg/ml, Abcam, UK), followed by incubation of biotinylated goat anti-mouse secondary antibody (Lab Vision Corporation, USA) or horseradish peroxidase (HRP)-conjugated anti-rabbit secondary antibody (Abcam), respectively.

### ECM and DNA quantification

Samples were first digested with 10 mg/ml of pepsin (Sigma-Aldrich) in 0.05 M acetic acid, followed by digestion with 1 mg/ml of elastase (Sigma-Aldrich). sGAG was quantified using the Blyscan sGAG assay kit (Biocolor, UK). The absorbance of sGAG in the samples was measured at 656 nm, and its concentration was extrapolated from the sGAG standard curve. Type II collagen (COL2) was measured via a captured enzyme-linked immunosorbent assay (ELISA; Chondrex, USA). Absorbance was measured at 490 nm, and the concentration of COL2 in samples was extrapolated from the COL2 standard curve. The total abundance of sGAG and COL2 were normalized to the total DNA content of respective samples, quantified by PicoGreen DNA assay.

### Western blot analysis

Samples were trypsinized and lysed using M-PER™ Mammalian protein extraction reagent (Thermo Fisher Scientific), supplemented with 1x Pierce™ Protease and Phosphatase Inhibitor (Thermo Fisher Scientific). The concentration of protein was quantified via Pierce™ BCA protein assay kit (Thermo Fisher Scientific). An equal amount of protein was electrophoresed on 12% 10-well Mini-PROTEAN® TGX™ Precast gel (Bio-Rad) and transferred onto a nitrocellulose membrane. The membranes were then incubated with the following antibodies: rabbit anti-ERK1 (phospho T202) + ERK2 (phospho T185) antibody (1 μg/ml; Abcam, UK; ab201015), rabbit anti-FAK (phospho Y576 + Y577) antibody (0.182 μg/ml; Abcam; ab76244), mouse anti-ERK1 + ERK2 antibody (1 μg/ml; Abcam; ab54230), mouse anti-FAK antibody (1 μg/ml; Abcam; ab72140), and mouse anti-β-actin antibody (1 μg/ml; Abcam; ab8226). Blots were subsequently incubated with respective HRP-conjugated secondary antibodies (1:5000 dilution), and immune complexes were then detected with SuperSignal® West Dura Extended Duration Substrate (Thermo Scientific) and viewed by ChemiDoc™ MP Imaging system (Bio-Rad). Densitometric analysis of the Western blot bands was then conducted using ImageJ software.

### Statistical analysis

Statistical significance was calculated after the data were tested for normality. One-way ANOVA followed by Fisher’s LSD post hoc analysis was performed for comparison of multiple groups, while Student’s *t* test was performed for comparison of two groups. All quantitative data obtained in this study were averaged from replicates of three independent experiments and presented in mean ± standard deviation (SD), with statistical significance of *p* < 0.05.

## Results

### Characterization of electrospun fiber sheets and MSC culture

**Fig. 2:**
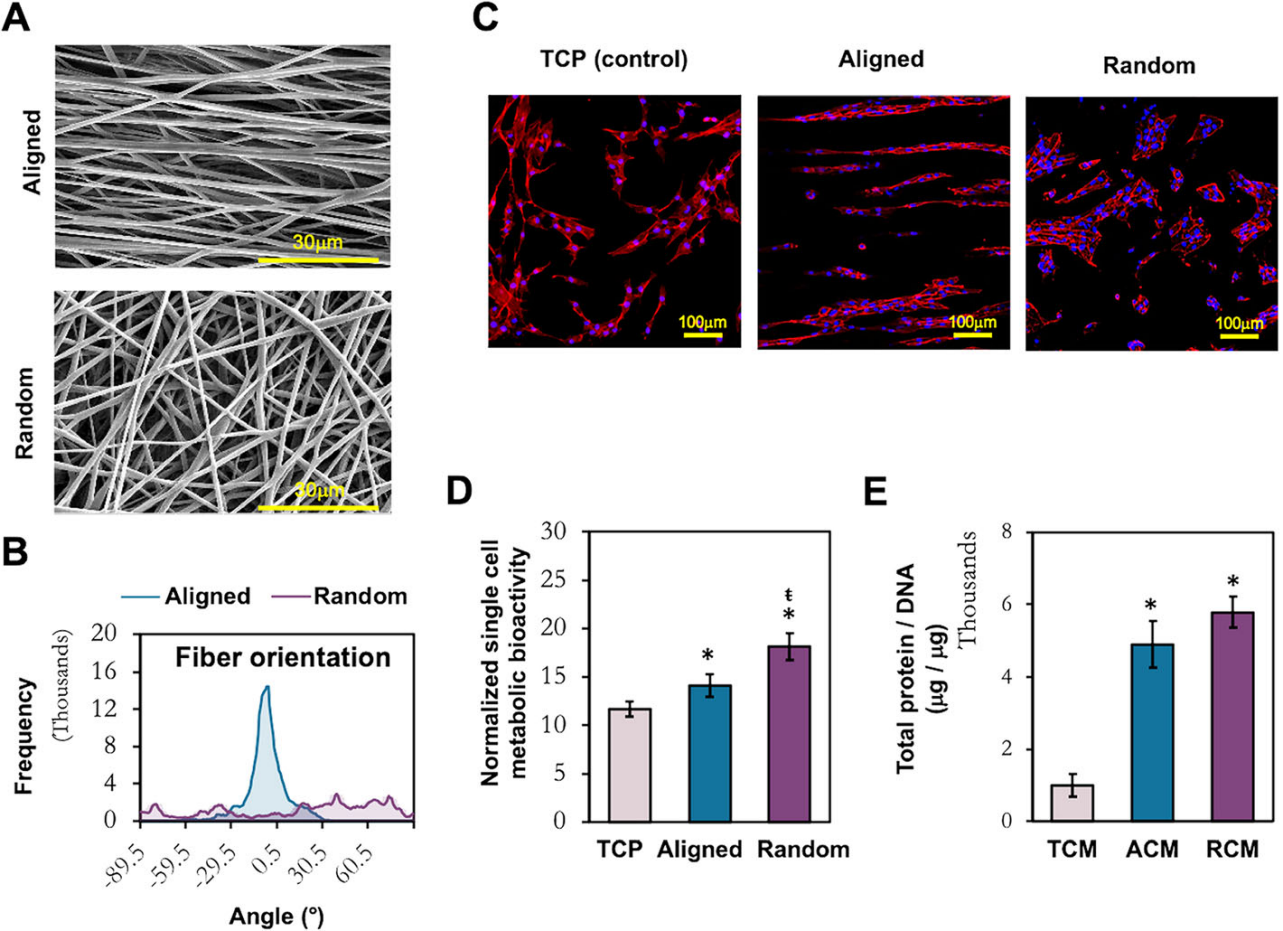
Characterization of electrospun fiber sheets and the adherent MSCs. **a** SEM images of the electrospun fiber sheets. Scale bar = 30 μm. **b** Analysis of fiber orientation. **c** Morphological evaluation of MSCs cultured on TCP, aligned and random fibers. Scale bar = 100 μm (× 200 magnification). **d** Metabolic activity of MSCs at the collection time point of CM. **e** Quantification of secreted protein in the CMs, normalized to the total cellular DNA of the conditioned MSCs. All data represent the mean ± SD, *n* = 6, from 3 independent experiments. * denotes *p* < 0.05 compared to TCP; ŧ denotes *p* < 0.05 compared to aligned fibers

SEM images revealed that PLCL electrospun fiber sheets were successfully fabricated with the desired topography (Fig. 2a) as indicated by the distinct fiber orientation between aligned and randomly oriented fiber sheets (Fig. 2b). Both aligned and randomly oriented PLCL fibers showed consistent fiber diameters and similar scaffold thickness and porosity (Table 1).

**Table 1:** Characteristic of 10% PLCL electrospun fibrous scaffolds of aligned and randomly oriented fibers

	Aligned	Random
Diameter (nm)	656.86 ± 40.94	653.60 ± 36.09
Thickness (μm)	101.72 ± 5.54	102.75 ± 13.16
% of porosity	11.79 ± 0.32	13.74 ± 0.65

MSCs cultured on different topographical fiber sheets formed distinct morphology in comparison to those on TCP (Fig. 2c). MSCs adopted spindle-like shape arranged along the direction of aligned fibers, compared to the fibroblastic morphology in a random arrangement on TCP. In contrast, the majority of MSCs on random fiber sheets formed aggregated clusters. MSCs conditioned on the fiber sheets have enhanced metabolic activity compared to TCP, with random fibers supporting the highest metabolically active MSCs (Fig. 2d). A higher yield of total protein content (> 5-fold) was detected in the CM derived from MSCs cultured on fiber sheets compared to TCP (Fig. 2e).

### Homing and reparative effect of ACM and RCM on chondrocytes and MSCs

**Fig. 3:**
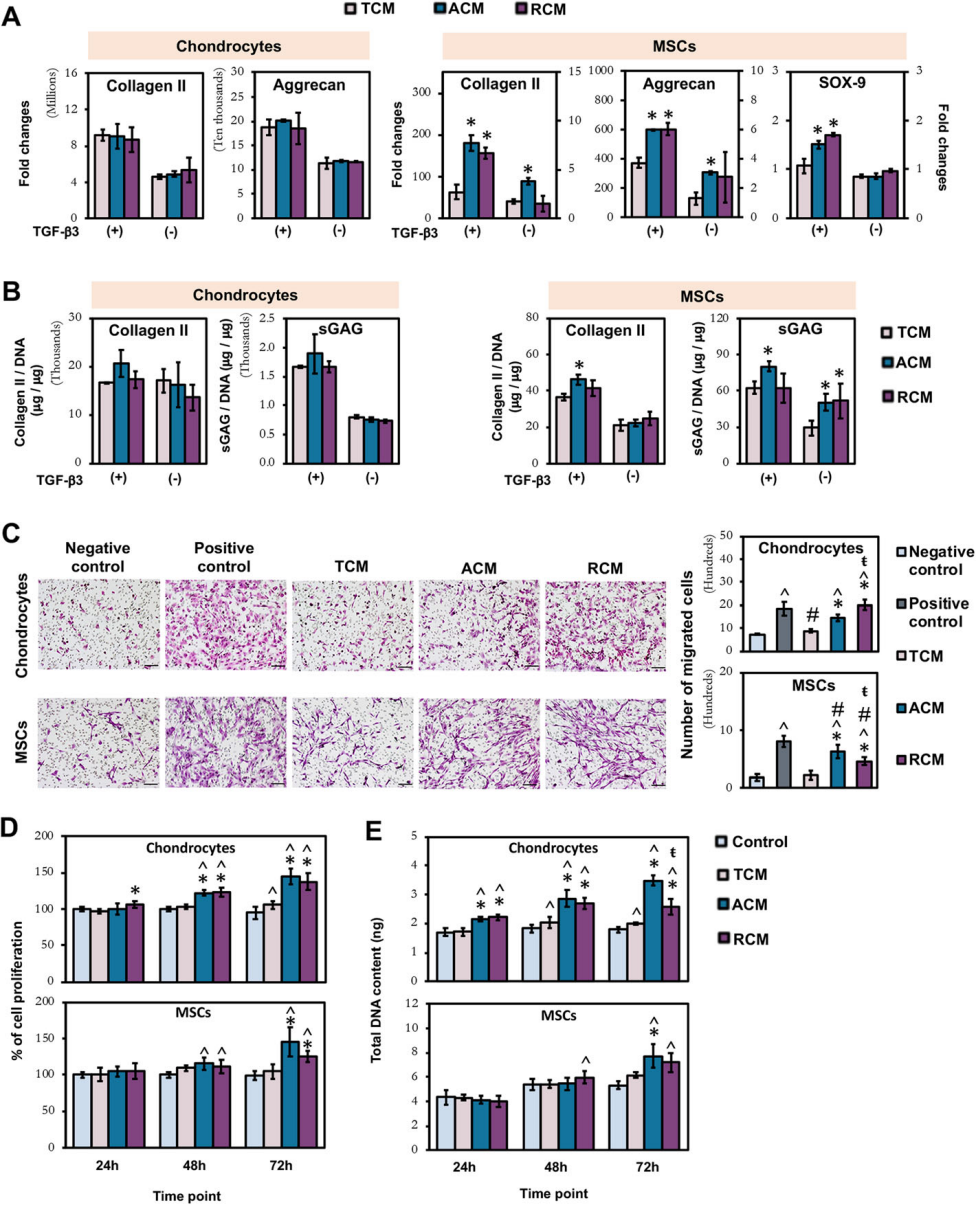
Effect of topographically induced CM on chondrogenesis (**a**, **b**), migration (**c**), and proliferation (**d**, **e**) of chondrocytes and MSCs. **a** Real-time PCR analysis of chondrogenic markers expression after 7 days of chondrogenic re-differentiation. **b** Quantification of cartilaginous ECM macromolecules after 14 (chondrocytes) and 21 days (MSCs) of differentiation. **c** Chondrocyte and MSC migration were assessed by transwell migration assay. Scale bar = 100 μm. **d** Quantification of chondrocyte and MSC proliferation and **e** total cellular DNA content. All data represent the mean ± SD, *n* = 6, from 3 independent experiments. ^ denotes *p* < 0.05 compared to control / negative control; # denotes *p* < 0.05 compared to positive control; * denotes *p* < 0.05 compared to TCM; ŧ denotes *p* < 0.05 compared to ACM

The chondrogenic, migratory, and proliferative potential of MSC-CM generated from electrospun fiber sheets (ACM and RCM) was compared to TCP-generated CM (TCM) on both chondrocytes and MSCs. ACM induced a consistent upregulation of COL2 and aggrecan in MSCs at both mRNA (Fig. 3a) and protein (Fig. 3b) levels with respect to TCM-treated MSCs. Comparatively, the enhancing effect of RCM was less consistent, in which increase of COL2, aggrecan, and SOX-9 mRNA expressions was only detected in the presence of TGF-β3 (Fig. 3a), while upregulation of sGAG deposition was only detected in the absence of TGF-β3 (Fig. 3b). Unlike augmentation to chondrogenic differentiation of MSCs, there was no enhancing effect of ACM and RCM relative to TCM for the re-differentiation of chondrocytes at both mRNA and protein levels (Fig. 3a, b).

Transwell migration study showed that ACM and RCM significantly enhanced the migration of both chondrocytes and MSCs relative to their respective negative controls and TCM (Fig. 3c). RCM induced the highest migratory capacity of chondrocytes, with migration level approaching the positive control (10% FBS), while ACM induced the highest migratory capacity of MSCs (Fig. 3c). By contrast, TCM induced limited (chondrocyte) or no (MSC) increase in migration relative to the negative control.

MTS (Fig. 3d) and DNA quantification (Fig. 3e) assays showed that chondrocytes and MSCs exposed to ACM and RCM were more proliferative than cells exposed to TCM and controls (0.5% FBS), with ACM exerting higher impact. However, both ACM and RCM exerted a greater proliferative effect on chondrocytes than MSCs, with significant enhancement in the percentage of chondrocyte proliferation (Fig. 3d) and DNA content (Fig. 3e) as early as 24 h. TCM did not induce enhanced proliferation in both cell types.

Overall, MSC secretome generated from distinct fiber orientations enhanced MSC chondrogenesis, chondrocyte, and MSC migration, as well as the proliferation of both cell types.

### Protective effect of ACM and RCM on chondrocytes and MSCs from inflammation assault and apoptosis

**Fig. 4:**
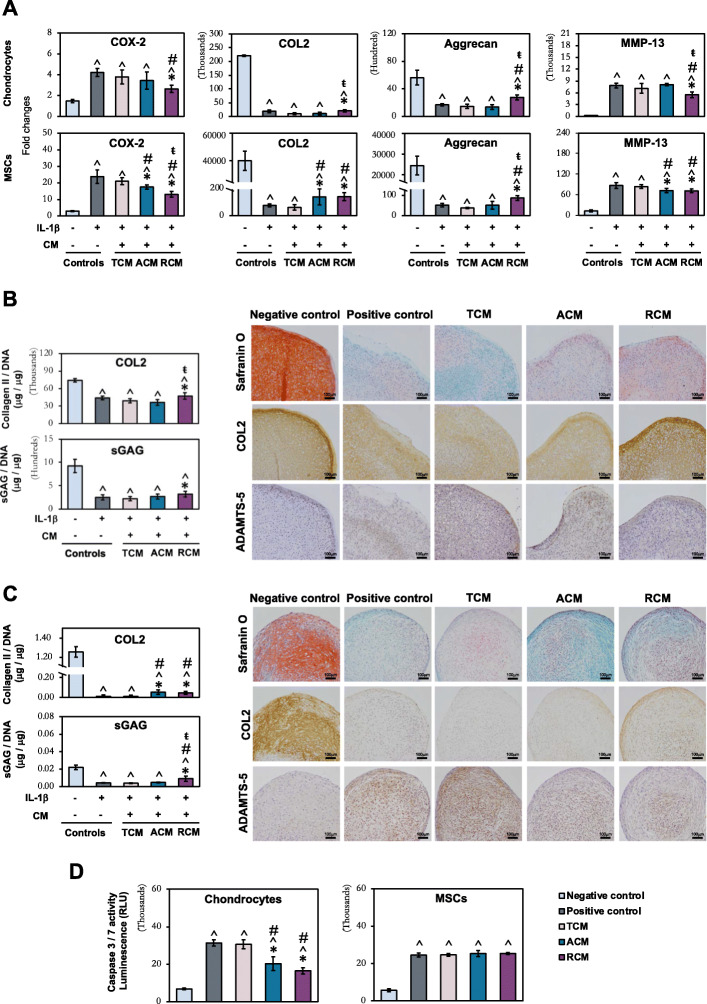
Inflammation modulatory (**a**–**c**) and anti-apoptotic (**d**) effect of topographically induced CM on chondrocytes and MSCs. **a** Real-time PCR analysis of inflammatory-related and chondrogenic markers expression in re-differentiated chondrocyte pellet and chondrogenic MSC pellet after 7 days of inflammation treatment with 5 ng/ml and 0.5 ng/ml IL-1β, respectively. Quantification and histological analysis of cartilaginous ECM macromolecules in **b** re-differentiated chondrocyte pellet after 7 days of inflammation treatment with 5 ng/ml IL-1β, and **c** MSC pellet after 21 days of chondrogenic induction under inflammation treatment of 0.5 ng/ml IL-1β. **d** Apoptotic activity of chondrocytes and MSCs as determined by Caspase 3/7 activity. All data represent the mean ± SD, *n* = 6, from 3 independent experiments. ^ denotes *p* < 0.05 compared to control / negative control; # denotes *p* < 0.05 compared to positive control; * denotes *p* < 0.05 compared to TCM; ŧ denotes *p* < 0.05 compared to ACM. Scale bar = 100 μm

The cellular protective function of MSC secretome has been attributed to its anti-inflammatory [11, 12, 28–30] and anti-apoptotic properties [11, 14]. We assessed the potential of CM to protect chondrocytes and MSCs from inflammation assault in IL-1β-induced inflammation models. The cellular protective effect of CM on inflammation-induced ECM degradation was assessed in re-differentiated chondrocyte tissues, while their effect on inflammation-induced chondrogenic inhibition was evaluated in MSCs undergoing chondrogenesis. The significantly high level of COX-2 expression in IL-1β-treated cells compared to non-treated cells showed that inflammation had been successfully induced in chondrocytes and MSCs (Fig. 4a).

In chondrocytes, a rescued effect by RCM on chondrocyte ECM production under inflammation relative to TCM and ACM was observed at both mRNA (Fig. 4a) and protein (Fig. 4b) levels. RCM-treated chondrocytes resulted in a slight, albeit significant, upregulation of COL2 and sGAG production, as well as downregulation of the aggrecanase, ADAMTS-5 (Fig. 4b) and MMP-13 (Fig. 4a), coinciding with a significant downregulation of COX-2 expression as observed only by RCM treatment (Fig. 4a).

For MSCs, both ACM and RCM rescued the differentiating MSCs under inflammation, with an increase ECM deposition of COL2 and aggrecan at both mRNA (Fig. 4a) and protein (Fig. 4c) levels, as well as a decrease in ADAMTS-5 (Fig. 4c) and MMP-13 (Fig. 4a) expression, relative to the TCM-treated MSC pellets. Again, the effect on the anabolic and catabolic activities of differentiating MSCs coincided with downregulation of COX-2 by both ACM and RCM treatment (Fig. 4a). Notably, RCM exerted greater rescued effect than ACM, with a further increment of aggrecan mRNA expression (Fig. 4a) and sGAG deposition (Fig. 4c), in parallel with the greater suppression of COX-2 expression.

We next investigated the anti-apoptotic potential of ACM and RCM on both chondrocytes and MSCs in staurosporine-induced apoptosis model. Treatment with ACM and RCM rescued the apoptotic chondrocytes with a significant reduction of caspase 3/7 activities relative to TCM, which has no effect on staurosporine (Positive control)-induced upregulation of caspase 3/7 activity (Fig. 4d). Unlike chondrocytes, apoptotic MSCs were not rescued by all three CM treatments (Fig. 4d).

Overall, our results indicate that orientation of fibers influences MSC secretome, which ameliorate inflammation in chondrocytes and MSCs by mitigating inflammation-induced ECM degradation and chondrogenic inhibition. Further, electrospun fiber-generated secretome reduced the progression of chondrocyte apoptosis.

### Expression and quantification of MSC secretome

**Fig. 5:**
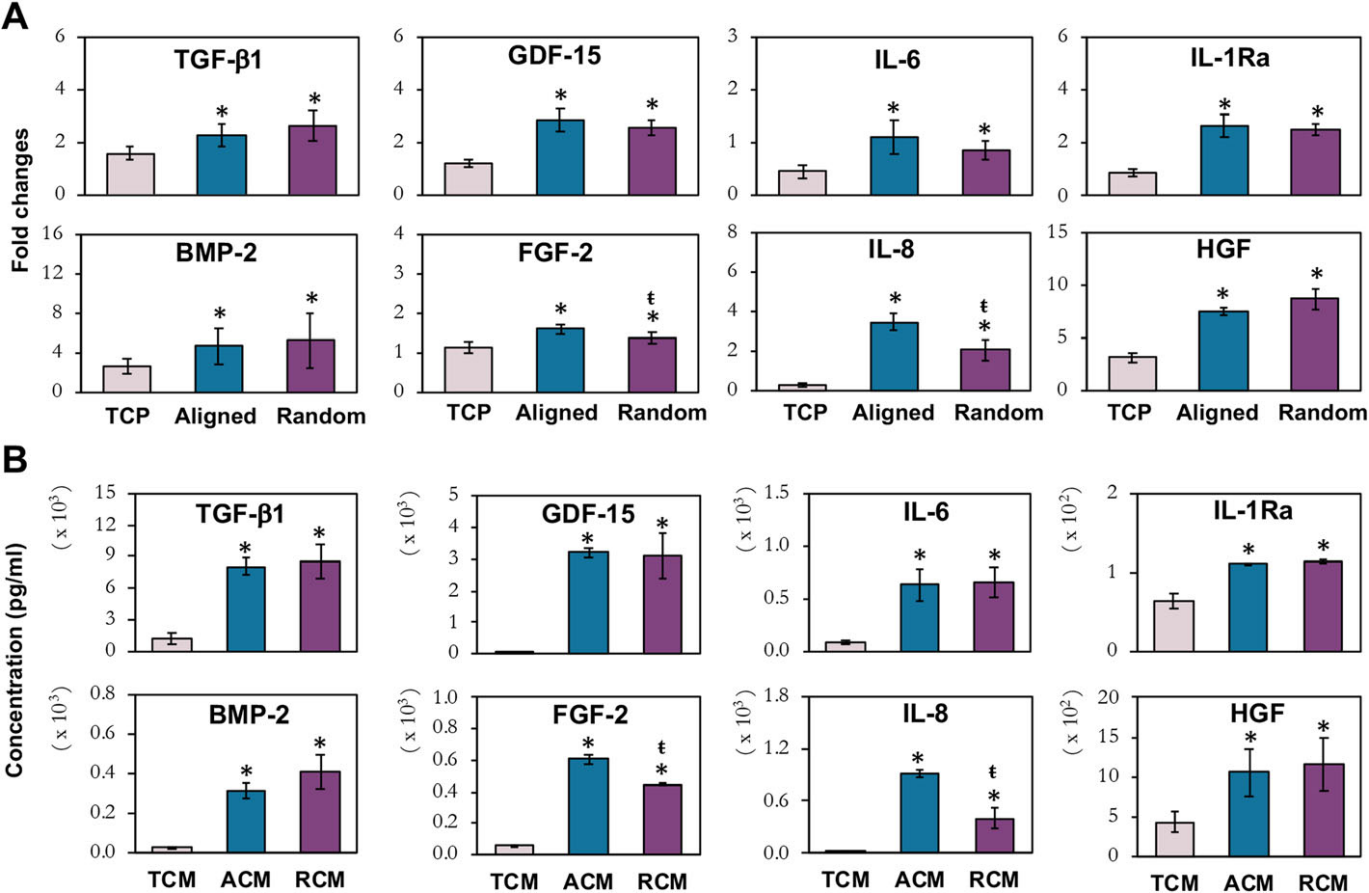
Characterization of MSC paracrine factors. mRNA expression (**a**) and protein concentration (**b**) of secretory factors in MSCs cultured on TCP, aligned and randomly oriented fiber sheets. **a** Real-time PCR data represent the mean ± SD, *n* ≥ 6 per group, from 3 independent experiments. * denotes *p* < 0.05 compared to MSCs cultured on TCP; ŧ denotes *p* < 0.05 compared to MSCs cultured on aligned fibers. **b** Luminex assay data represent the mean ± SD, *n* = 4 per group, from 2 MSC donors. * denotes *p* < 0.05 compared to TCM; ŧ denotes *p* < 0.05 compared to ACM

The influence of fiber orientation associated with their positive paracrine effects on chondrogenic differentiation, migration, proliferation, and inflammatory modulation was further investigated by analyzing the expression of MSC secretory factors. We detected upregulation of TGF-β1, BMP-2, GDF-15, FGF-2, IL-6, IL-8, IL-1Ra, and HGF mRNA in MSCs cultured on aligned and randomly oriented fibers, relative to TCP (Fig. 5a). Amongst the upregulated factors, FGF-2 and IL-8 levels were significantly higher in aligned fibers than randomly oriented fibers. A Luminex assay was performed on TCM, ACM, and RCM to characterize the secretome profile. The enhanced expression of secretory factors, TGF-β1 (6-fold relative to TCM), BMP-2 (> 12-fold), GDF-15 (> 100-fold), FGF-2 (> 7-fold), IL-6 (8-fold), IL-8 (> 15-fold), IL-1Ra (1.7-fold), and HGF (> 2-fold) in ACM and RCM (Fig. 5b) corroborated well with the mRNA expression in MSCs preconditioned on the fiber sheets (Fig. 5a). Similarly, ACM has significantly higher levels of FGF-2 and IL-8 than RCM. The parallel observation of enhanced secretory factors in the fiber sheet-generated CM along with the increased mRNA expressions in MSCs cultured on electrospun fiber sheets suggests that changes in MSC morphology and mechano-environment induced by fiber orientation have altered the MSC secretome profile.

### Activation of FAK signaling by electrospun fiber sheets

**Fig. 6:**
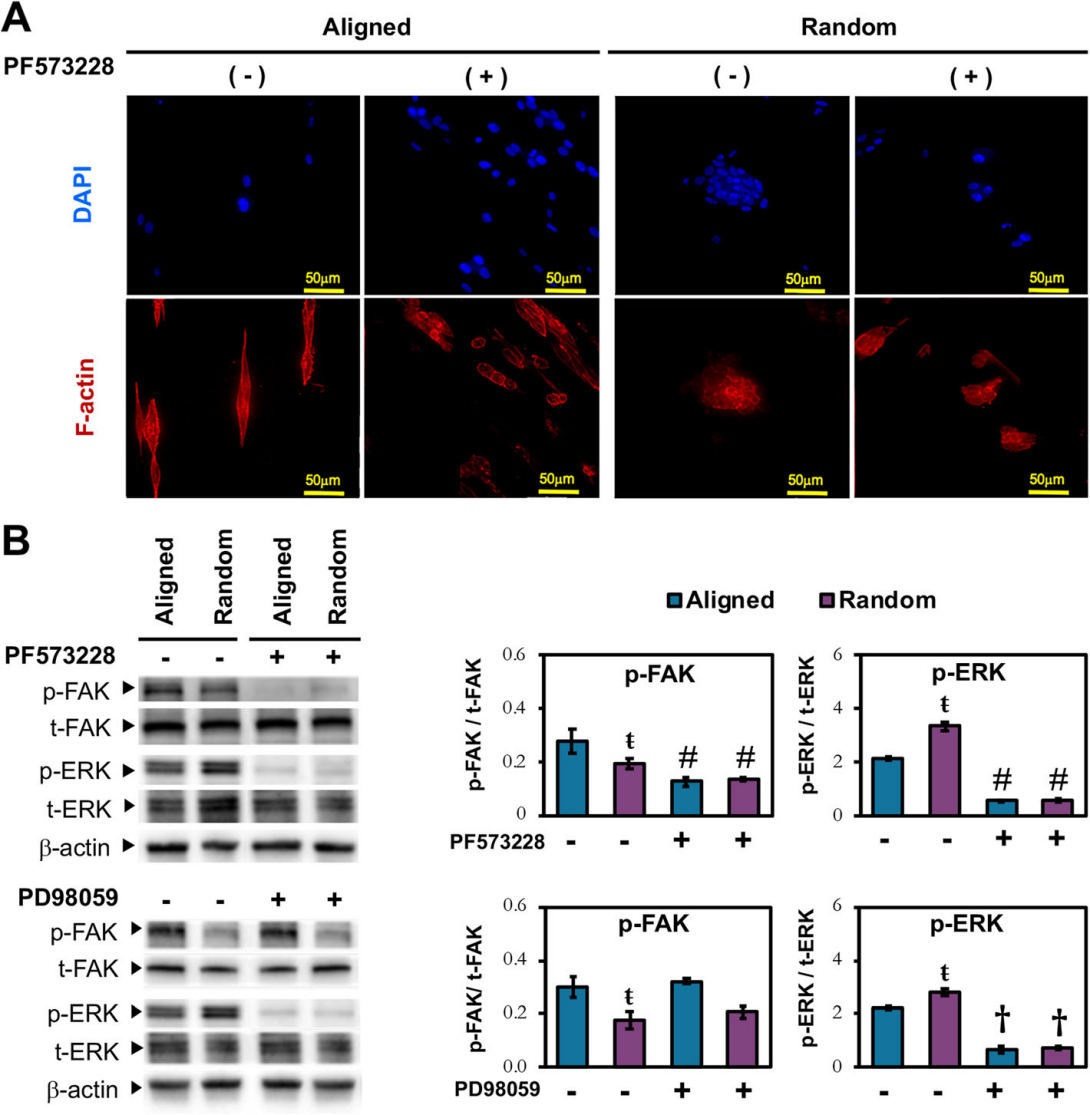
Association of FAK / ERK signaling pathways to MSC morphology on electrospun fiber sheets. **a** Phalloidin staining of F-actin (red) and nuclei with DAPI (blue) in MSCs treated with or without FAK inhibitor (PF573228). Scale bar = 50 μm. **b** Western blot and densitometric analysis of phosphorylated FAK (p-FAK) and ERK (p-ERK) in MSCs cultured on aligned and random fibers in the absence or presence of FAK inhibitor (20 μM; PF573228) or ERK inhibitor (70 μM; PD98059) for 24 h. Densitometric analysis of p-FAK and p-ERK were normalized to their respective non-phosphorylated counterpart. All data represent mean ± SD, *n* = 6, from 3 independent experiments. ŧ denotes *p* < 0.05 compared to aligned fibers; † denotes *p* < 0.05 compared to MSCs without PD98059 treatment; # denotes *p* < 0.05 compared to MSCs without PF573228 treatment

Morphological change of MSCs and the activation of mechanotransduction pathways are associated with the response of MSCs to topographical cues, in which FAK is a key component recruited at the focal contact of cell-matrix adhesion [31]. Activation of FAK triggers downstream mitogen-activated protein kinase (MAPK) pathway, with ERK as the key link that regulates the cellular response to mechanotransduction stimuli [32]. We thus further explored the expression of activated FAK and ERK along with the cytoskeleton reorganization of MSCs preconditioned on aligned and randomly oriented fibers. MSCs cultured on aligned fibers possessed F-actin stress fibers (Fig. 6a), with higher FAK activation than MSCs on randomly oriented fibers (Fig. 6b). By contrast, MSCs conditioned on randomly oriented fibers expressed cortical actin (Fig. 6a), while having a higher level of ERK activation (Fig. 6b). Inhibition of FAK further confirmed its involvement in cell-matrix adhesion as the suppressed p-FAK expression (Fig. 6b) led to a change in F-actin organization, with the transition from a more prominently spindled to a rounder morphology for MSCs cultured on aligned fibers (Fig. 6a). FAK inhibition also resulted in suppression of p-ERK expression in MSCs cultured on both fiber sheets, regardless of its orientation (Fig. 6b). ERK inhibition, on the other hand, did not affect p-FAK expression (Fig. 6b), indicating that FAK activation is upstream of ERK pathway. These inhibition studies confirmed that the orientation of fibers could influence MSC cytoskeletal structure and FAK-induced ERK activation.

### Influence of FAK and ERK activation in modulating MSC paracrine factors

**Fig. 7:**
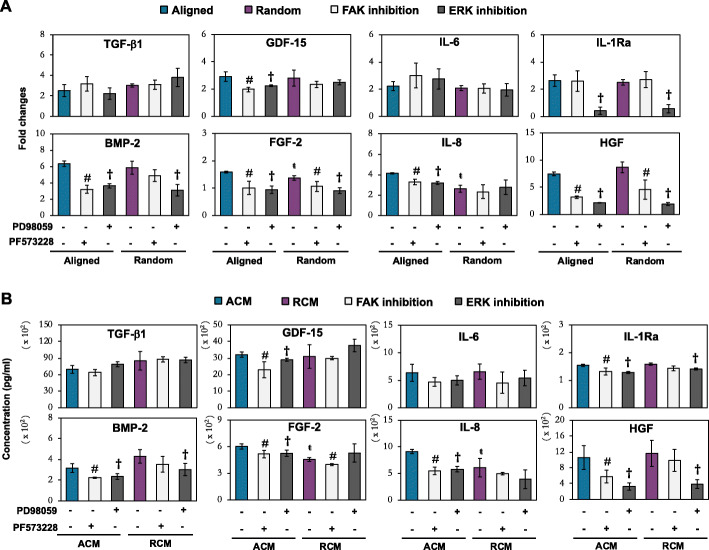
Effect of FAK and ERK inhibition in **a** fiber-conditioned MSCs and **b** its protein secretory profiles. MSCs were treated with FAK (PF573228; 20 μM) and ERK (PD98059; 70 μM) inhibitors. **a** Real-time PCR data represent the mean ± SD, *n* = 6 per group, from 3 independent experiments. **b** Luminex assay data represent the mean ± SD, *n* = 4 per group, from 2 MSC donors. ŧ denotes *p* < 0.05 compared to the aligned fibers. # denotes *p* < 0.05 between MSC treated with PF573228 and their respective non-treated MSCs; † denotes *p* < 0.05 between MSC treated with PD98059 and their respective non-treated MSCs

To investigate the role of FAK and ERK activation in MSCs’ secretome generation on electrospun fiber sheets, we evaluated the expression of the upregulated paracrine factors after inhibition of either FAK (PF573228) or ERK (PD98059) signaling pathways (Fig. 7). The enhanced expression of BMP-2, FGF-2, GDF-15, IL-8, and HGF in MSCs conditioned on aligned fibers were significantly reduced upon treatment with either FAK or ERK inhibitors at both mRNA (Fig. 7a) and secreted protein levels (Fig. 7b). However, MSCs cultured on randomly oriented fibers only showed significant suppression of FGF-2 upon FAK inhibition, while the expression of HGF and BMP-2 were only suppressed upon ERK inhibition (Fig. 7a, b). A similar trend of dampened effect was also observed in IL-1Ra secreted protein levels, in which MSCs conditioned on aligned fibers showed a significant reduction of IL-1Ra expression upon FAK or ERK inhibition, while MSCs conditioned on random fibers showed suppression of IL-1Ra only upon ERK inhibition (Fig. 7b). The lack of statistical significance in the reduction of IL-8 and GDF-15 expression by both FAK and ERK inhibitors in MSCs conditioned on randomly oriented fibers (Fig. 7a), suggested that these factors were regulated by other signaling cascades, in relation to the change of MSC morphology. Lastly, the lack of statistical significance in the expression of TGF-β1 and IL-6 after inhibition of FAK or ERK pathways on both aligned and random fibers (Fig. 7a, b), suggested that these factors were not directly regulated by FAK or ERK signaling cascades.

## Discussion

Provision of native-mimicking microenvironment has been recognized as a critical factor to improve the therapeutic efficacy of MSCs for cartilage formation [33–36] and secretome production [16, 20, 23, 29]. Morphological variation in MSCs acquired through cell-substratum and cell-cell interaction can be manipulated to exert beneficial paracrine effects, such as promoting cell migration and proliferation [20], as well as modulating inflammation [25, 26]. Electrospun fibrous scaffolds have been particularly attractive as they provide a fibrillar architecture that distinctively influences MSC-substratum interaction and activates mechanotransduction signaling cascade, leading to a change in MSC secretome profile [26]. In this study, we investigated the ability of CM generated from MSCs cultured on electrospun fiber sheets with a distinct fiber orientation, relative to those generated from TCP, to promote homing, proliferation, reparation, and protection of chondrocytes, as well as MSCs, the resident stem cells in the vicinity of a synovial joint that participate in cartilage regeneration [37].

Collectively, the enhanced paracrine functional outcomes of electrospun fiber-generated CM (ACM and RCM) relative to TCM correlated with their enhanced level of secreted factors that have known migratory, proliferative, chondrogenic, and immunomodulation functions. The comparable upregulation of TGF-β1 and BMP-2 [38, 39], the two well reported chondro-inducing factors, in MSCs and their secretion upon subjected to aligned and random fiber sheets (Fig. 5) could account for the augmented MSC chondrogenesis (Fig. 3a, b). The higher efficacy of ACM to augment MSC chondrogenesis compared to RCM could be attributed by the presence of other secreted factors, such as the elevated level of IL-8, which has been shown to enhance MSC chondrogenesis [40]. IL-8 has multiple functions that include MSC migration [40, 41]; together with the elevated levels of IL-6 [42] and HGF [43, 44] in fiber sheet-generated secretome (Fig. 5), this could have contributed to the increased migratory potential in ACM and RCM (Fig. 3c). The heightened level of TGFβ1, FGF-2, and GDF-15 in ACM and RCM (Fig. 5b), on the other hand, could have contributed to the increased proliferation in chondrocytes and MSCs (Fig. 3d, e). TGFβ1 and FGFs are growth factors that induced MSC proliferation [45, 46] and are commonly used for chondrocyte expansion [47, 48]. GDF-15, a MSC secretory product [49] belonging to the BMP family, has also been associated with cell proliferation [50]. Notably, the significant higher levels of FGF in ACM relative to RCM (Fig. 5) were correlated with the heightened proliferative potential of ACM-treated cells (Fig. 3d, e). This observation was in accordance with Su et al. (2017), in which MSCs was reported to secrete greater amount of FGF-2 on aligned than random fibers [29]. Notably, the proliferative effect of MSC secretome appeared to be more effective on chondrocytes than MSCs (Fig. 3d, e). This prominent effect on chondrocytes could possibly be due to the presence of HGF in the CM as this growth factor has been shown to promote chondrocyte proliferation [43], but was however inhibitive towards MSC proliferation [44]. Thus, a collective synergistic effect of elevated factors such as TGF-β1, BMP-2, FGF-2, GDF-15, HGF, IL-6, and IL-8 in ACM and RCM could have potentiated the chondrogenic, proliferation, and recruitment capacities towards chondrocytes and MSCs. Nevertheless, it is also noteworthy that other factor(s) not tested within the very limited number of screened proteins could also participate in augmenting the paracrine effects, such as the capability of MSC-derived EVs in promoting the migration and proliferation of chondrocytes [11, 51].

Following cartilage injury or in osteoarthritis, degenerative, and apoptotic joint environment are perpetuated as the heightened level of pro-inflammatory cytokines and catabolic factors suppressed the chondrocyte anabolic activities [52]. MSC secretome has been reported to alleviate the inflammatory environment of cartilage and impede inflammation-induced ECM cartilage degradation [11–13, 28, 30]. Here, we showed that CM from fiber sheets significantly downregulated the inflammation status of chondrocytes and MSCs, dampened the catabolic activities and reverted, to some extent, the anabolic activities (Fig. 4a–c), as well as rescued the staurosporine-induced chondrocytes apoptosis (Fig. 4d). The protective effect of the fiber-generated CM was correlated to the elevated secretion of IL-1Ra, HGF, and GDF-15 (Fig. 5). Intra-articular injection of IL-1Ra, an IL1-β antagonist, has been shown to delay cartilage degradation in arthritic joint [53], while HGF ameliorated inflammation through inhibition of NF-ĸB pathway [54], and GDF-15 modulated inflammation with extended cellular protection against apoptosis [55]. Despite having similar levels of IL-1Ra, HGF, and GDF-15, RCM holds heightened protective capability from inflammation assault in chondrocytes and MSCs, evidenced by the greater COX-2, MMP-13 (Fig. 4a), and ADAMTS-5 suppression, with concomitant improvement of ECM deposition (Fig. 4b, c) and cellular protection against staurosporine-induced chondrocytes apoptosis (Fig. 4d), relative to ACM. It is thus likely that other factor(s), such as exosomal microRNAs, could have participated in regulating chondrocyte anabolic activity and cartilage degradation [56, 57].

Contrary to several previous reports [11, 14, 28, 30], secretome derived from MSCs cultured on TCP in our study did not induce enhanced migration and proliferation, nor provide protection against inflammation to chondrocytes. The discrepancy in the efficacy of TCP-derived secretome could stem from the use of different cell sources [11, 58, 59]. However, closer examination of the protocols employed by others revealed that their secretome (CM, EVs, or exosomes) were generated from a higher quantity of MSCs [11, 30, 60] compared to the CM used in this study. Strikingly, the secretome from our low number of MSCs on fibrous culture platform was able to exert significant paracrine effects. ACM or RCM treatments induced > 2-fold increase in chondrocyte migration relative to the negative control or TCM (Fig. 3c), an effect comparable in magnitude with 10 μg/ml of exosome treatment [11]. In addition, the degree of enhanced chondrocyte proliferation at 72 h time point (~ 1.6-fold increase) in ACM-treated chondrocytes (Fig. 3d, e) was also comparable to the chondrocytes treated with 5 μg/ml exosomes at 72 h (~ 1.6-fold increase) [11]. It is also worth mentioning that the secretome from our low number of MSCs on fibrous culture platform could provide protection against an adverse inflammatory condition, exerting significant, albeit slight, rescued effect (Fig. 4a–c). We speculated that the rescued effect would likely be more effective with CM dosage generated from higher quantity of MSCs in the similar range as other studies [11, 30]. The enhanced paracrine effects of fiber-generated CM that corroborated with the increase in total secretory proteins (≥ 5-fold relative to TCM; Fig. 2e) and specific secretory factors (Fig. 5b) could also stem from the heightened status of MSC metabolic activity on fibrous culture platforms (Fig. 2d). Metabolically active stromal cells were shown to secrete higher levels of cytokines with an enhanced immunomodulatory capacity [61]. Thus, conditioning of MSCs on fibrous culture platform holds great potential over the current conventional TCP for improving the yield and repertoire of MSC secretome for cartilage repair.

The variation in the degree of paracrine effects between ACM and RCM however suggests the presence of potentially topographical-dependent MSC secretome. Substrate stiffness and topographical cues induced a change in cell morphology via the cells’ ability to sense the magnitude of traction force [62] that activates intracellular mechanotransduction pathways [63, 64]. FAK is one of the early signaling proteins recruited at the focal contact of cell-matrix adhesion through the activation of integrin receptors [65], which in turn activated ERK1/2, a MAPK associated with substrate stiffness or topography-induced mechanotransduction signaling [26, 66]. Similar to Wan et al. [26], we observed that the heightened FAK activation in MSCs subjected to the aligned fiber sheets (Fig. 6b) corresponded to the elevation of cellular tension, as indicated by the presence of F-actin stress fibers (Fig. 2c). In contrast, the heightened level of p-FAK activation in MSCs on aligned fibers did not correspond to the higher levels of downstream ERK1/2 activation relative to MSCs cultured on randomly oriented fibers (Fig. 6b). The discrepancy in relative ERK1/2 activation could be attributed to the distinct traction force experienced by MSCs when subjected to different scaffolding materials. A stiff and inflexible poly-L-lactic acid (PLLA) scaffolds were employed in Wan et al. study, in which MSCs formed polygonal morphology with F-actin stress fibers on the random scaffold [26]. Unlike PLLA scaffolds, the more compliant elastomeric PLCL fiber sheets and the relatively high cell seeding density used in current study has allowed MSCs to experience less traction force and enhanced cell-cell interaction that promote cell agglomeration on random fibers (Fig. 2c). By analogy, the metabolically more active MSCs on random fibers relative to the aligned fibers (Fig. 2d) could be attributed to the formation of agglomerated MSCs as actin-mediated cellular compaction reprogramed and induced MSC energy metabolism [67, 68].

**Fig. 8:**
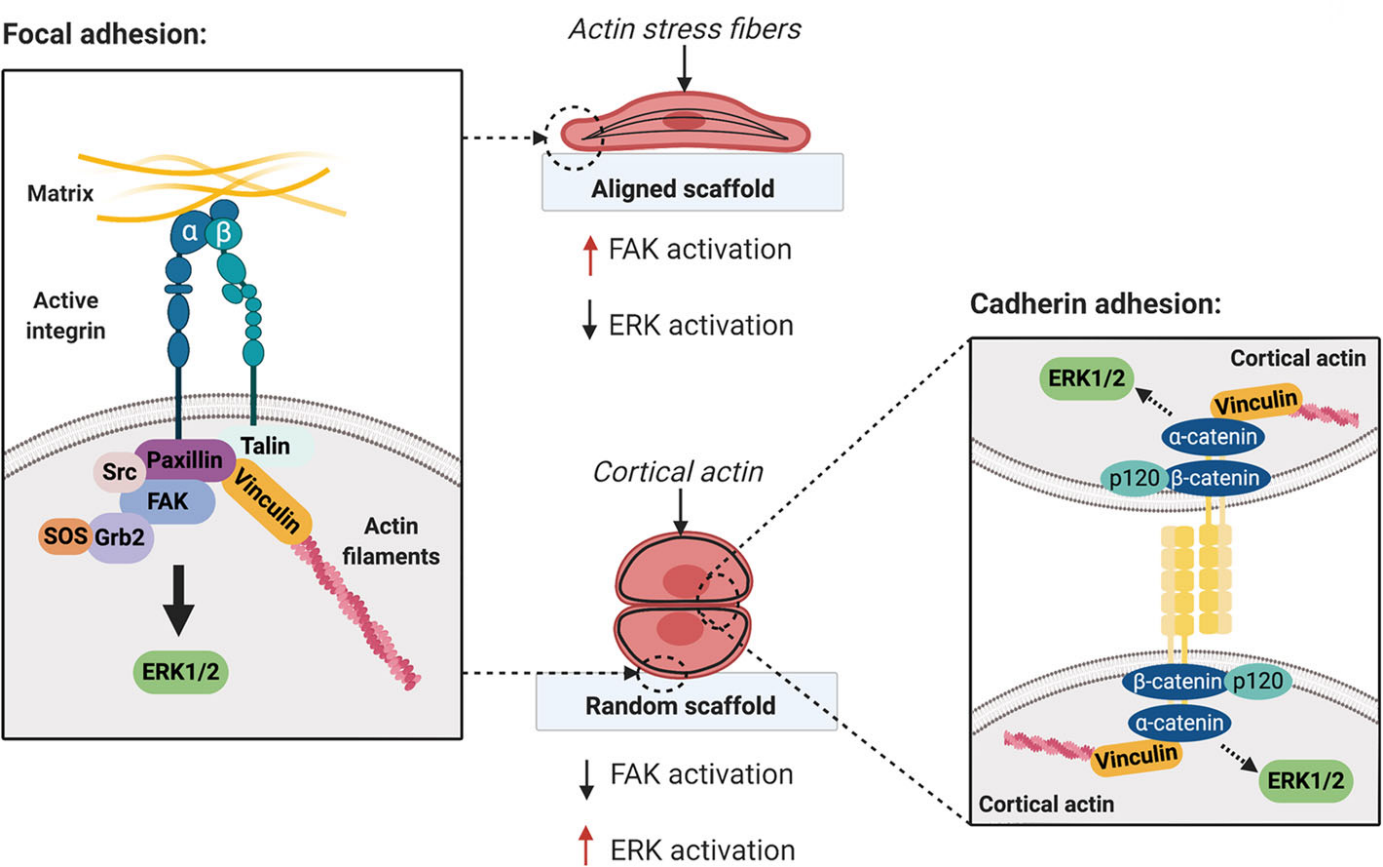
The possible underlying mechanisms in aligned and random fibers via FAK and ERK signaling cascades. Created with BioRender.com

Inhibition of FAK or ERK pathways revealed that fiber orientation could modulate the repertoire of MSC secretome through the extensiveness of FAK/ERK activation (Fig. 7). In general, the enhanced paracrine effects of MSCs induced by aligned fiber sheets required FAK-induced ERK pathways. This was supported by the significant reduction of BMP-2, GDF-15, FGF-2, IL-8, IL-1Ra, and HGF expression and secretion upon either FAK or ERK inhibition. Indeed, the association of FAK activation and the enhanced secretion of these proteins in morphologically stretched MSCs on the aligned fibers are in accordance to the reported mechanotransduction regulation of BMP-2 [69], HGF [70], IL-1Ra [71], GDF-15 [72], IL-8 [73], and FGF-2 [25]. Although these secretory factors were similarly augmented on the random fiber sheets, its regulation in agglomerated MSCs was more complex and differed from the stretched MSCs on aligned fiber sheets. FGF-2 expressed in MSCs on random fibers was affected by inhibition of FAK, but not ERK, while IL-8 and GDF-15 expression was neither affected by FAK nor ERK inhibitors, while expressions of BMP-2, HGF, and IL-1Ra in MSCs were regulated by ERK-dependent pathway. Aggregated or spheroid MSCs have been shown to enhance the secretion of several cytokines and growth factors [74], via cadherin-dependent cell-cell interaction [20, 75, 76] that triggered downstream ERK1/2 activation [75, 76]. MSC mechano-conditioned on aligned and randomly oriented fibers could thus activate distinct mechanotransduction pathway, through FAK and / or ERK signaling (Fig. 8). The secretory factors derived from stretched MSCs on aligned fibers were predominantly regulated via the FAK-ERK pathway, while agglomerated MSCs on random fibers could have triggered the ERK signaling pathway via cadherin induction, accounting for comparable expression of MSC paracrine factors induced by the two distinctive fiber orientation. However, given the partial effect of either FAK or ERK inhibition on the abovementioned factors, and the lack of effect on increased expression of TGF-β1 and IL-6, additional signaling pathways were likely involved in the regulation of topographical-dependent MSC secretome. A broader analysis of altered genes and signaling pathways using technology such as RNA-Seq could further shed light in the understanding of the paracrine factors and mechanotransduction mechanism triggered by the fiber orientation on MSCs and the future strategies utilizing MSC secretome for in vivo application.

## Conclusions

In this study, we have provided evidence that preconditioning of MSCs on both aligned and random fiber sheets can enhance their ability to secrete paracrine factors with the capability to promote MSC chondrogenesis, migration, and proliferation of MSCs and chondrocytes, as well as provide protection against an adverse inflammatory condition. Mechano-conditioning on distinct fiber orientations triggered different FAK-ERK signaling pathways and exerted variable paracrine effects, suggestive of the potential presence of topographical-dependent MSC secretome. Application of RCM could prevent worsening of the pre-existing articular cartilage as it was more efficient in mitigating the inflammation assault. In contrast, ACM could aid in repairing and restoring the function of the damaged cartilage as it was better at promoting cell proliferation and augmenting MSC chondrogenesis. This research has made a significant contribution to the development of MSC secretome engineering strategies for cartilage regeneration, as well as the understanding of cell-material interactions in improving MSC secretome for future cell-free therapy in cartilage regeneration.

## Supplementary Information

**Additional file 1: Figure S1.** Dosage effect of IL-1β to induce inflammation in (A) chondrocytes and (B) MSC chondrogenic pellets. (A) Inflammation was induced in chondrocytes either with 0, 1, 5 or 10 ng/ml of IL-1β. Real-time PCR analysis after 24 h induction of IL-1β was normalized to GAPDH and presented as fold change relative to the level in non-treated (Day 0) chondrocytes. (B) Inflammation was induced in MSC chondrogenic pellet either with 0, 0.5, 1 or 2 ng/ml of IL-1β. Real-time PCR analysis after 7 days of chondrogenesis under the continuous presence of IL-1β-induced inflammation, was normalized to GAPDH and presented as fold change relative to the level in undifferentiated MSCs. All data represent the mean ± standard deviation (STD), *n* = 6, from 3 independent experiments. ^ denotes *p* < 0.05 compared to untreated cells (0 ng/ml of IL-1β); * denotes p < 0.05 compared to cells treated with 1 ng/ml of IL-1β in chondrocytes or 0.5 ng/ml of IL-1β in MSC pellets; # denotes p < 0.05 between chondrocytes treated with 10 ng/ml and 5 ng/ml of IL-1β.

**Additional file 2: Table S1.** Primer sequences of genes.

## Data Availability

All data generated during this study are included in this published article and its supplementary information files.

## Abbreviations

ACM: Aligned fiber-generated conditioned media
ADAMTS-5: ADAM metallopeptidase with thrombospondin type 1 motif 5
BMP-2: Bone morphogenetic proteins-2
CM: Conditioned media
COL2: Type II collagen
COX-2: Cyclooxygenase-2
DMEM: Dulbecco’s modified Eagle’s medium
ECM: Extracellular matrix
ELISA: Enzyme-linked immunosorbent assay
ERK: Extracellular signal-regulated kinase
FAK: Focal adhesion kinase
FGF-2: Fibroblast growth factors
GDF-15: Growth differentiation factors 15
HGF: Hepatocyte growth factor
HRP: Horseradish peroxidase
IL-1β: Interleukin-1β
IL-6: Interleukin-6
IL-8: Interleukin-8
MAPK: Mitogen-activated protein kinase
MSCs: Mesenchymal stem cells
MTS: 3-(4,5-Dimethylthiazol-2-yl)-5-(3-carboxymethoxyphenyl)-2-(4-sulfophenyl)-2H- tetrazolium
PCR: Polymerase chain reaction
PLCL: Poly L-lactide-co-ε-caprolactone
PLLA: Poly-L-lactic acid
RCM: Randomly oriented fiber-generated conditioned media
SEM: Scanning electron microscope
sGAG: Sulfated glycosaminoglycan
SOX-9: Sex-determining region Y-box 9
TCM: TCP-generated conditioned media
TCP: Tissue culture plate
TGF-β1: Transforming growth factor-β1
TGF-β3: Transforming growth factor-β3

## References

[1] Gobbi A, Karnatzikos G, Kumar A (2014). Long-term results after microfracture treatment for full-thickness knee chondral lesions in athletes. Knee Surg Sport Traumatol Arthrosc.

[2] Kim YS, Choi YJ, Lee SW, Kwon OR, Suh DS, Heo DB (2016). Assessment of clinical and MRI outcomes after mesenchymal stem cell implantation in patients with knee osteoarthritis: a prospective study. Osteoarthr Cartil.

[3] Shin Y-S, Yoon J-R, Kim H-S, Lee S-H. Intra-articular injection of bone marrow-derived mesenchymal stem cells leading to better clinical outcomes without difference in MRI outcomes from baseline in patients with knee osteoarthritis. Knee Surg Relat Res 2018;30(3):206–14. Available from: http://www.ncbi.nlm.nih.gov/pubmed/29983008. [cited 2019 Oct 26] 10.5792/ksrr.17.201 PMC6122947 29983008

[4] Goldring MB, Otero M (2011). Inflammation in osteoarthritis. Curr Opin Rheumatol.

[5] Keeney M, Lai JH, Yang F (2011). Recent progress in cartilage tissue engineering. Curr Opin Biotechnol.

[6] Nejadnik H, Hui JH, Feng Choong EP, Tai B-C, Lee EH (2010). Autologous bone marrow–derived mesenchymal stem cells versus autologous chondrocyte implantation. Am J Sports Med.

[7] Toh WS (2017). The emerging role of mesenchymal stem cell secretome in cartilage regeneration. Front Stem Cell Regen Med Res.

[8] Angulski ABB, Correa A, Stimamiglio MA. Mesenchymal stem/stromal cells as biological factories. In: Mesenchymal stromal cells as tumor stromal modulators Elsevier; 2017. p. 121–54. Available from: https://linkinghub.elsevier.com/retrieve/pii/B9780128031025000057. [cited 2019 Oct 7]

[9] Wu J, Kuang L, Chen C, Yang J, Zeng WN, Li T, et al. miR-100-5p-abundant exosomes derived from infrapatellar fat pad MSCs protect articular cartilage and ameliorate gait abnormalities via inhibition of mTOR in osteoarthritis. Biomaterials. 2019;206:87–100. 10.1016/j.biomaterials.2019.03.022. 10.1016/j.biomaterials.2019.03.022 30927715

[10] Zhang S, Chu WC, Lai RC, Lim SK, Hui JHP, Toh WS (2016). Exosomes derived from human embryonic mesenchymal stem cells promote osteochondral regeneration. Osteoarthr Cartil.

[11] Zhang S, Chuah SJ, Lai RC, Hui JHP, Lim SK, Toh WS (2018). MSC exosomes mediate cartilage repair by enhancing proliferation, attenuating apoptosis and modulating immune reactivity. Biomaterials.

[12] Cosenza S, Ruiz M, Toupet K, Jorgensen C, Noël D (2017). Mesenchymal stem cells derived exosomes and microparticles protect cartilage and bone from degradation in osteoarthritis. Sci Rep.

[13] Wang Y, Yu D, Liu Z, Zhou F, Dai J, Wu B (2017). Exosomes from embryonic mesenchymal stem cells alleviate osteoarthritis through balancing synthesis and degradation of cartilage extracellular matrix. Stem Cell Res Ther.

[14] Chen W, Sun Y, Gu X, Hao Y, Liu X, Lin J (2019). Conditioned medium of mesenchymal stem cells delays osteoarthritis progression in a rat model by protecting subchondral bone, maintaining matrix homeostasis, and enhancing autophagy. J Tissue Eng Regen Med.

[15] Zavatti M, Beretti F, Casciaro F, Bertucci E, Maraldi T (2020). Comparison of the therapeutic effect of amniotic fluid stem cells and their exosomes on monoiodoacetate-induced animal model of osteoarthritis. BioFactors..

[16] Kusuma GD, Carthew J, Lim R, Frith JE (2017). Effect of the microenvironment on mesenchymal stem cell paracrine signaling: opportunities to engineer the therapeutic effect. Stem Cells Dev.

[17] Hsu W-T, Lin C-H, Chiang B-L, Jui H-Y, Wu KK-Y, Lee C-M (2013). Prostaglandin E _2_ potentiates mesenchymal stem cell–induced IL-10 ^+^ IFN-γ ^+^ CD4 ^+^ regulatory T cells to control transplant arteriosclerosis. J Immunol.

[18] Xia P, Wang X, Qu Y, Lin Q, Cheng K, Gao M, et al. TGF-β1-induced chondrogenesis of bone marrow mesenchymal stem cells is promoted by low-intensity pulsed ultrasound through the integrin-mTOR signaling pathway. Stem Cell Res Ther. 2017;8(281):1-11. 10.1186/s13287-017-0733-9. 10.1186/s13287-017-0733-9 PMC5729425 29237506

[19] YlÖstalo JH, Bartosh TJ, Coble K, Prockop DJ (2012). Human mesenchymal stem/stromal cells cultured as spheroids are self-activated to produce prostaglandin E2 that directs stimulated macrophages into an anti-inflammatory phenotype. Stem Cells.

[20] Qazi TH, Mooney DJ, Duda GN, Geissler S. Biomaterials that promote cell-cell interactions enhance the paracrine function of MSCs. Biomaterials. 2017;140:103–114. Available from: http://www.ncbi.nlm.nih.gov/pubmed/28644976. [cited 2019 Nov 17] 10.1016/j.biomaterials.2017.06.019 28644976

[21] DeLise AM, Fischer L, Tuan RS (2000). Cellular interactions and signaling in cartilage development. Osteoarthr Cartil.

[22] Goldring MB, Tsuchimochi K, Ijiri K (2006). The control of chondrogenesis. J Cell Biochem.

[23] Jose S, Hughbanks ML, Binder BYK, Ingavle GC, Leach JK (2014). Enhanced trophic factor secretion by mesenchymal stem/stromal cells with Glycine-Histidine-Lysine (GHK)-modified alginate hydrogels. Acta Biomater.

[24] Yang H, Nguyen KT, Leong DT, Tan NS, Tay CY. Soft material approach to induce oxidative stress in mesenchymal stem cells for functional tissue repair. ACS Appl Mater Interfaces. 2016;8(40):26591–9. 10.1021/acsami.6b09222. 10.1021/acsami.6b09222 27608498

[25] Su N, Gao P-L, Wang K, Wang J-Y, Zhong Y, Luo Y (2017). Fibrous scaffolds potentiate the paracrine function of mesenchymal stem cells: A new dimension in cell-material interaction. Biomaterials.

[26] Wan S, Fu X, Ji Y, Li M, Shi X, Wang Y (2018). FAK- and YAP/TAZ dependent mechanotransduction pathways are required for enhanced immunomodulatory properties of adipose-derived mesenchymal stem cells induced by aligned fibrous scaffolds. Biomaterials.

[27] Zhang T, Wen F, Wu Y, Goh G, Ge Z, Tan L (2015). Cross-talk between TGF-beta/SMAD and integrin signaling pathways in regulating hypertrophy of mesenchymal stem cell chondrogenesis under deferral dynamic compression. Biomaterials.

[28] Platas J, Guillén MI, Del Caz MDP, Gomar F, Mirabet V, Alcaraz MJ. Conditioned media from adipose-tissue-derived mesenchymal stem cells downregulate degradative mediators induced by interleukin-1 β in osteoarthritic chondrocytes. Mediat Inflamm. 2013;2013:1-10. 10.1155/2013/357014. 10.1155/2013/357014 PMC3864089 24363499

[29] Su N, Gao P-L, Wang K, Wang J-Y, Zhong Y, Luo Y. Fibrous scaffolds potentiate the paracrine function of mesenchymal stem cells: A new dimension in cell-material interaction. Biomaterials. 2017;141:74–85. Available from:http://www.ncbi.nlm.nih.gov/pubmed/28667901. [cited 2019 Oct 4] 10.1016/j.biomaterials.2017.06.028 28667901

[30] Vonk LA, van Dooremalen SFJ, Liv N, Klumperman J, Coffer PJ, Saris DBF (2018). Mesenchymal stromal/stem cell-derived extracellular vesicles promote human cartilage regeneration in vitro. Theranostics.

[31] Gregor M, Osmanagic-Myers S, Burgstaller G, Wolfram M, Fischer I, Walko G (2014). Mechanosensing through focal adhesion-anchored intermediate filaments. FASEB J.

[32] Shih Y-R V, Tseng K-F, Lai H-Y, Lin C-H, Lee OK (2011). Matrix stiffness regulation of integrin-mediated mechanotransduction during osteogenic differentiation of human mesenchymal stem cells. J Bone Miner Res.

[33] Raghothaman D, Leong MF, Lim TC, Toh JKC, Wan ACA, Yang Z, et al. Engineering cell matrix interactions in assembled polyelectrolyte fiber hydrogels for mesenchymal stem cell chondrogenesis. Biomaterials. 2014;35(9):2607–16. 10.1016/j.biomaterials.2013.12.008. 10.1016/j.biomaterials.2013.12.008 24388815

[34] Wu Y-N, Law JBK, He AY, Low HY, Hui JHP, Lim CT (2014). Substrate topography determines the fate of chondrogenesis from human mesenchymal stem cells resulting in specific cartilage phenotype formation. Nanomedicine.

[35] Baker BM, Nathan AS, Gee AO, Mauck RL. The influence of an aligned nanofibrous topography on human mesenchymal stem cell fibrochondrogenesis. Biomaterials. 2010;31(24):6190–200. Available from: http://www.ncbi.nlm.nih.gov/pubmed/20494438. [cited 2019 Oct 7] 10.1016/j.biomaterials.2010.04.036 PMC2884056 20494438

[36] Shafiee A, Soleimani M, Chamheidari GA, Seyedjafari E, Dodel M, Atashi A (2011). Electrospun nanofiber-based regeneration of cartilage enhanced by mesenchymal stem cells. J Biomed Mater Res Part A.

[37] Zhang W, Chen J, Tao J, Jiang Y, Hu C, Huang L (2013). The use of type 1 collagen scaffold containing stromal cell-derived factor-1 to create a matrix environment conducive to partial-thickness cartilage defects repair. Biomaterials.

[38] Shen B, Wei A, Tao H, Diwan AD, Ma DDF (2009). BMP-2 enhances TGF-β3-mediated chondrogenic differentiation of human bone marrow multipotent mesenchymal stromal cells in alginate bead culture. Tissue Eng - Part A.

[39] Xia P, Wang X, Qu Y, Lin Q, Cheng K, Gao M (2017). TGF-β1-induced chondrogenesis of bone marrow mesenchymal stem cells is promoted by low-intensity pulsed ultrasound through the integrin-mTOR signaling pathway. Stem Cell Res Ther.

[40] Yang A, Lu Y, Xing J, Li Z, Yin X, Dou C (2018). IL-8 enhances therapeutic effects of BMSCs on bone regeneration via CXCR2-mediated PI3k/Akt signaling pathway. Cell Physiol Biochem.

[41] Mishima Y, Lotz M (2008). Chemotaxis of human articular chondrocytes and mesenchymal stem cells. J Orthop Res.

[42] Pricola KL, Kuhn NZ, Haleem-Smith H, Song Y, Tuan RS (2009). Interleukin-6 maintains bone marrow-derived mesenchymal stem cell stemness by an ERK1/2-dependent mechanism. J Cell Biochem.

[43] Takebayashi T, Iwamoto M, Jikko A, Matsumura T, Enomoto-Iwamoto M, Myoukai F (1995). Hepatocyte growth factor/scatter factor modulates cell motility, proliferation, and proteoglycan synthesis of chondrocytes. J Cell Biol.

[44] Forte G, Minieri M, Cossa P, Antenucci D, Sala M, Gnocchi V, et al. Hepatocyte growth factor effects on mesenchymal stem cells: proliferation, migration, and differentiation article in stem cells · cell and gene therapy for hemophilia A View project Biomimetic approximation to the magnetosome biomineralization process in magnetotactic bacteria. CGL2016–76723-P View project Hepatocyte Growth Factor Effects on Mesenchymal Stem Cells: Proliferation, Migration, and Differentiation. 2006; Available from: http://www.miltenyibiotec.com. [cited 2020 May 4]

[45] Ng F, Boucher S, Koh S, Sastry KSR, Chase L, Lakshmipathy U, et al. PDGF, tgf- 2. And FGF signaling is important for differentiation and growth of mesenchymal stem cells (mscs): transcriptional profiling can identify markers and signaling pathways important in differentiation of MSCs into adipogenic, chondrogenic, and osteogenic lineages. Blood. 2008;112(2):295–307. Available from: www.ingenuity.com. [cited 2020 Aug 5] 10.1182/blood-2007-07-103697 18332228

[46] Rodrigues M, Griffith LG, Wells A. Growth factor regulation of proliferation and survival of multipotential stromal cells. Stem Cell Res Ther. 2010;1(32):1–12. 10.1186/scrt32. 10.1186/scrt32 PMC2983445 20977782

[47] Yonekura A, Osaki M, Hirota Y, Tsukazaki T, Miyazaki Y, Matsumoto T (1999). Transforming growth factor-.BETA. Stimulates articular chondrocyte cell growth through p44/42 MAP kinase (ERK) activation. Endocr J.

[48] Francioli SE, Martin I, Sie CP, Hagg R, Tommasini R, Candrian C, et al. Growth factors for clinical-scale expansion of human articular chondrocytes: relevance for automated bioreactor systems. Tissue Eng. 2007;13(6):1227–34.10.1089/ten.2006.0342. 10.1089/ten.2006.0342 17518725

[49] Kim DH, Lee D, Chang EH, Kim JH, Hwang JW, Kim JY, et al. GDF-15 secreted from human umbilical cord blood mesenchymal stem cells delivered through the cerebrospinal fluid promotes hippocampal neurogenesis and synaptic activity in anAlzheimer’s disease model. Stem Cells Dev. 2015;24(20):2378–90. 10.1089/scd.2014.0487. 10.1089/scd.2014.0487 PMC4598918 26154268

[50] Uchiyama T, Kawabata H, Miura Y, Yoshioka S, Iwasa M, Yao H (2015). The role of growth differentiation factor 15 in the pathogenesis of primary myelofibrosis. Cancer Med.

[51] Zhu Y, Wang Y, Zhao B, Niu X, Hu B, Li Q (2017). Comparison of exosomes secreted by induced pluripotent stem cell-derived mesenchymal stem cells and synovial membrane-derived mesenchymal stem cells for the treatment of osteoarthritis. Stem Cell Res Ther.

[52] Kapoor M, Martel-Pelletier J, Lajeunesse D, Pelletier JP, Fahmi H. Role of proinflammatory cytokines in the pathophysiology of osteoarthritis. Nat Rev Rheumatol. 2011;7(1):33–42. 10.1038/nrrheum.2010.196. 10.1038/nrrheum.2010.196 21119608

[53] Elsaid KA, Ubhe A, Shaman Z, D’Souza G. Intra-articular interleukin-1 receptor antagonist (IL1-ra) microspheres for posttraumatic osteoarthritis: in vitro biological activity and in vivo disease modifying effect. J Exp Orthop. 2016;3(18):1–10.10.1186/s40634-016-0054-4. 10.1186/s40634-016-0054-4 PMC4990523 27539076

[54] Bendinelli P, Matteucci E, Dogliotti G, Corsi MM, Banfi G, Maroni P (2010). Molecular basis of anti-inflammatory action of platelet-rich plasma on human chondrocytes: mechanisms of NF-κB inhibition via HGF. J Cell Physiol.

[55] Heger J, Schiegnitz E, von Waldthausen D, Anwar MM, Piper HM, Euler G. Growth differentiation factor 15 acts anti-apoptotic and pro-hypertrophic in adult cardiomyocytes. J Cell Physiol. 2010;224(1):n/a-n/a. Available from: http://www.ncbi.nlm.nih.gov/pubmed/20232299. [cited 2019 Dec 10] 10.1002/jcp.22102 20232299

[56] Wu J, Kuang L, Chen C, Yang J, Zeng W-N, Li T, et al. miR-100-5p-abundant exosomes derived from infrapatellar fat pad MSCs protect articular cartilage and ameliorate gait abnormalities via inhibition of mTOR in osteoarthritis. Biomaterials 2019;206:87–100. Available from: http://www.ncbi.nlm.nih.gov/pubmed/30927715. [cited 2019 Dec 31] 10.1016/j.biomaterials.2019.03.022 30927715

[57] Mao G, Hu S, Zhang Z, Wu P, Zhao X, Lin R (2018). Exosomal miR-95-5p regulates chondrogenesis and cartilage degradation via histone deacetylase 2/8. J Cell Mol Med.

[58] Roche S, D’Ippolito G, Gomez LA, Bouckenooghe T, Lehmann S, Montero-Menei CN (2013). Comparative analysis of protein expression of three stem cell populations: Models of cytokine delivery system in vivo. Int J Pharm.

[59] Pires AO, Mendes-Pinheiro B, Teixeira FG, Anjo SI, Ribeiro-Samy S, Gomes ED (2016). Unveiling the differences of secretome of human bone marrow mesenchymal stem cells, adipose tissue-derived stem cells, and human umbilical cord perivascular cells: a proteomic analysis. Stem Cells Dev.

[60] Lai RC, Yeo RWY, Padmanabhan J, Choo A, de Kleijn DPV, Lim SK (2016). Isolation and characterization of exosome from human embryonic stem cell-derived C-Myc-immortalized mesenchymal stem cells.

[61] Melief SM, Zwaginga JJ, Fibbe WE, Roelofs H (2013). Adipose tissue-derived multipotent stromal cells have a higher immunomodulatory capacity than their bone marrow-derived counterparts. Stem Cells Transl Med.

[62] Galbraith CG, Sheetz MP, Stamenovic D, Fredberg JJ, Mijailovich SM, Tolic-Norrelykke IM (1997). A micromachined device provides a new bend on fibroblast traction forces. Proc Natl Acad Sci.

[63] Teo BKK, Wong ST, Lim CK, Kung TYS, Yap CH, Ramagopal Y (2013). Nanotopography modulates mechanotransduction of stem cells and induces differentiation through focal adhesion kinase. ACS Nano.

[64] Dalby MJ, Gadegaard N, ROC O (2014). Harnessing nanotopography and integrin–matrix interactions to influence stem cell fate. Nat Mater.

[65] Vitillo L, Kimber SJ (2017). Integrin and FAK regulation of human pluripotent stem cells. Curr stem Cell Rep.

[66] Chaturvedi LS, Marsh HM, Basson MD (2007). Src and focal adhesion kinase mediate mechanical strain-induced proliferation and ERK1/2 phosphorylation in human H441 pulmonary epithelial cells. Am J Physiol Physiol.

[67] Bijonowski BM, Daraiseh SI, Yuan X, Ma T (2019). Size-dependent cortical compaction induces metabolic adaptation in mesenchymal stem cell aggregates. Tissue Eng - Part A.

[68] Tsai AC, Liu Y, Yuan X, Ma T (2015). Compaction, fusion, and functional activation of three-dimensional human mesenchymal stem cell aggregate. Tissue Eng - Part A.

[69] Zeng Z, Yin X, Zhang X, Jing D, Feng X (2015). Cyclic stretch enhances bone morphogenetic protein-2-induced osteoblastic differentiation through the inhibition of Hey1. Int J Mol Med.

[70] Tatsumi R, Hattori A, Ikeuchi Y, Anderson JE, Allen RE (2002). Release of hepatocyte growth factor from mechanically stretched skeletal muscle satellite cells and role of pH and nitric oxide. Mol Biol Cell.

[71] Lee RT, Briggs WH, Cheng GC, Rossiter HB, Libby P, Kupper T (1997). Mechanical deformation promotes secretion of IL-1 alpha and IL-1 receptor antagonist. J Immunol.

[72] Frank D, Kuhn C, Brors B, Hanselmann C, Lüdde M, Katus HA (2008). Gene expression pattern in biomechanically stretched cardiomyocytes: evidence for a stretch-specific gene program. Hypertens (Dallas, Tex 1979).

[73] Harada M, Osuga Y, Hirota Y, Koga K, Morimoto C, Hirata T (2005). Mechanical stretch stimulates interleukin-8 production in endometrial stromal cells: possible implications in endometrium-related events. J Clin Endocrinol Metab.

[74] Bartosh TJ, Ylostalo JH, Mohammadipoor A, Bazhanov N, Coble K, Claypool K (2010). Aggregation of human mesenchymal stromal cells (MSCs) into 3D spheroids enhances their antiinflammatory properties. Proc Natl Acad Sci.

[75] Lee EJ, Choi E-K, Kang SK, Kim G-H, Park JY, Kang H-J (2012). N-cadherin determines individual variations in the therapeutic efficacy of human umbilical cord blood-derived mesenchymal stem cells in a rat model of myocardial infarction. Mol Ther.

[76] Lee EJ, Park SJ, Kang SK, Kim G-H, Kang H-J, Lee S-W (2012). Spherical bullet formation via E-cadherin promotes therapeutic potency of mesenchymal stem cells derived from human umbilical cord blood for myocardial infarction. Mol Ther.

