# Supplementary figures and images for: Electrospun fibers enhanced the paracrine signaling of mesenchymal stem cells for cartilage regeneration
Source: Stem Cell Res Ther. 2021 Feb 3;12:100. doi: 10.1186/s13287-021-02137-8 (PMC7860031; doi:10.1186/s13287-021-02137-8)

**A**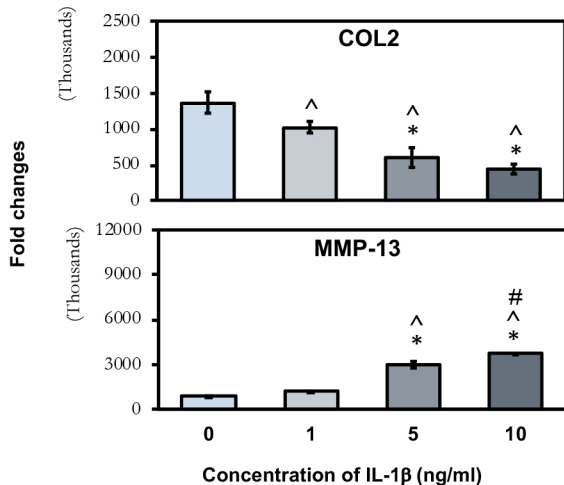**B**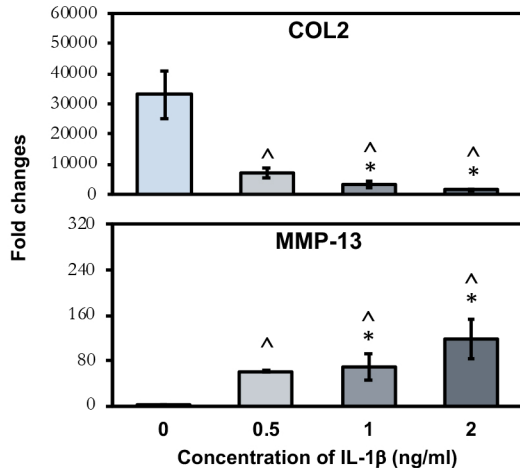

Supplement: Supplementary file 1 — Additional file 1: Figure S1. Dosage effect of IL-1β to induce inflammation in (A) chondrocytes and (B) MSC chondrogenic pellets. (A) Inflammation was induced in chondrocytes either with 0, 1, 5 or 10 ng/ml of IL-1β. Real-time PCR analysis after 24 h induction of IL-1β was normalized to GAPDH and presented as fold change relative to the level in non-treated (Day 0) chondrocytes. (B) Inflammation was induced in MSC chondrogenic pellet either with 0, 0.5, 1 or 2 ng/ml of IL-1β. Real-time PCR analysis after 7 days of chondrogenesis under the continuous presence of IL-1β-induced inflammation, was normalized to GAPDH and presented as fold change relative to the level in undifferentiated MSCs. All data represent the mean ± standard deviation (STD), n = 6, from 3 independent experiments. ^ denotes p < 0.05 compared to untreated cells (0 ng/ml of IL-1β); * denotes p < 0.05 compared to cells treated with 1 ng/ml of IL-1β in chondrocytes or 0.5 ng/ml of IL-1β in MSC pellets; # denotes p < 0.05 between chondrocytes treated with 10 ng/ml and 5 ng/ml of IL-1β. [file 13287_2021_2137_MOESM1_ESM.pdf]
