# Supplementary material for: Electrospun fibers enhanced the paracrine signaling of mesenchymal stem cells for cartilage regeneration
Source: Stem Cell Res Ther. 2021 Feb 3;12:100. doi: 10.1186/s13287-021-02137-8 (PMC7860031; doi:10.1186/s13287-021-02137-8)
Supplement: Supplementary file 2 — Additional file 2: Table S1. Primer sequences of genes. [file 13287_2021_2137_MOESM2_ESM.pdf]

| Species | Gene targets | Primer sequences | |
|---|---|---|---|
| Human | GDF-15 | Forward | 5’ –TCAAGGTCGTGGGACGTGACA-3’ |
| | | Reverse | 5’ – GCCGTGCGGACGAAGATTCT- 3’ |
| | IL-8 | Forward | 5’-GACCACACTGCGCCAACAC-3’ |
| | | Reverse | 5’-CTTCTCCACAACCCTCTGCAC-3’ |
| | COX-2 | Forward | 5’-CAGCACTTCACGCATCAGTTT-3’ |
| | | Reverse | 5’-GCGCAGTTTACGCTGTCTA-3’ |
| | IL-6 | Forward | 5’-GATGGCTGAAAAAGATGGATGC-3’ |
| | | Reverse | 5’-TGGTTGGGTCAGGGGTGGTT-3’ |
| | MMP-13 | Forward | 5’-TCCTCTTCTTGAGCTGGACTC-3’ |
| | | Reverse | 5’-CGCTCTGCAAACTGGAGGTC-3’ |
| | TGF-β1 | Forward | 5’-CGCATCCTAGACCCTTTCTCCTC-3’ |
| | | Reverse | 5’-GGTGTCTCAGTATCCCACGGAAAT-3’ |
| | BMP-2 | Forward | 5’-ATGGATTCGTGGTGGAAGTG -3’ |
| | | Reverse | 5’-GTGGAGTTCAGATGATCAGC -3’ |
| | FGF-2 | Forward | 5’-GGCTGTAGAACAAATGGCCT-3’ |
| | | Reverse | 5’-AGCCAACTCGTAACAATCCA-3’ |
| | HGF | Forward | 5’-CCCCATCGCCATCCCCTATG-3’ |
| | | Reverse | 5’-ACATCTATTAGCACATTGGTCTGCAGT-3’ |
| | IL-1Ra | Forward | 5’-AAGATGTGCCTGTCCTGTGTCAA-3’ |
| | | Reverse | 5’-GTTCTCGCTCAGGTCAGTGATGTTA-3’ |
| | GAPDH | Forward | 5’-ATGGGGAAGGTGAAGGTCG-3’ |
| | | Reverse | 5’-TAAAAGCAGCCCTGGTGACC-3’ |
| | COL2 | Forward | 5’-GGCAATAGCAGGTTCACGTACA-3’ |

| | | | |
|---|---|---|---|
| | | Reverse | 5’-CGATAACAGTCTTGCCCCACTT-3’ |
| | Aggrecan | Forward | 5’-ACTTC CGCTGGTCAGATGGA-3’ |
| | | Reverse | 5’-TCTCGTGCCAGATCATCACC-3’ |
| | SOX-9 | Forward | 5’-AGTACCCGCACTTGCACAA-3’ |
| | | Reverse | 5 ’-CTCGTTCAGAAGTCT CCAGAGCTT-3’ |
| Porcine | COX-2 | Forward | 5’-TTCAACCAGCAATTCCAATACCA -3’ |
| | | Reverse | 5’- GAAGGCGTCAGGCAGAAG-3’ |
| | MMP-13 | Forward | 5’-CTTGTTTCTTGTTGCTGCCC-3’ |
| | | Reverse | 5’-GTTGGGGTCTTCATCTCCTG-3’ |
| | GAPDH | Forward | 5’-ATGGTGAAGGTCGGAGTGAA-3’ |
| | | Reverse | 5’-AATGAAGGGGTCATTGATGG-3’ |
| | COL2 | Forward | 5’-TGAGAGGTCTTCCTGGCAAA-3’ |
| | | Reverse | 5’-GAAGTCCCTGGAAGCCAGAT-3’ |
| | Aggrecan | Forward | 5’-CATCACCGAGGGTGAAGC-3’ |
| | | Reverse | 5’-CCAGGGGCAAATGTAAAGG-3’ |
